# Supplementary material for: Nondestructive X-ray tomography of brain tissue ultrastructure
Source: Nat Methods. 2025 Nov 27;22(12):2631–8. doi: 10.1038/s41592-025-02891-0 (PMC12695642; doi:10.1038/s41592-025-02891-0)
Supplement: Supplementary file 1 — Supplementary Fig. 1. [file 41592_2025_2891_MOESM1_ESM.pdf]

nature methods

Supplementary information

https://doi.org/10.1038/s41592-025-02891-0

Nondestructive X-ray tomography of brain tissue ultrastructure

In the format provided by the authors and unedited

Supplementary Figures

Table of contents

Table of contents 0

Supplementary Figures 1

Supplementary Fig. 1: Extracellular space in samples containing olfactory bulb external plexiform layer 1

Supplementary Figures

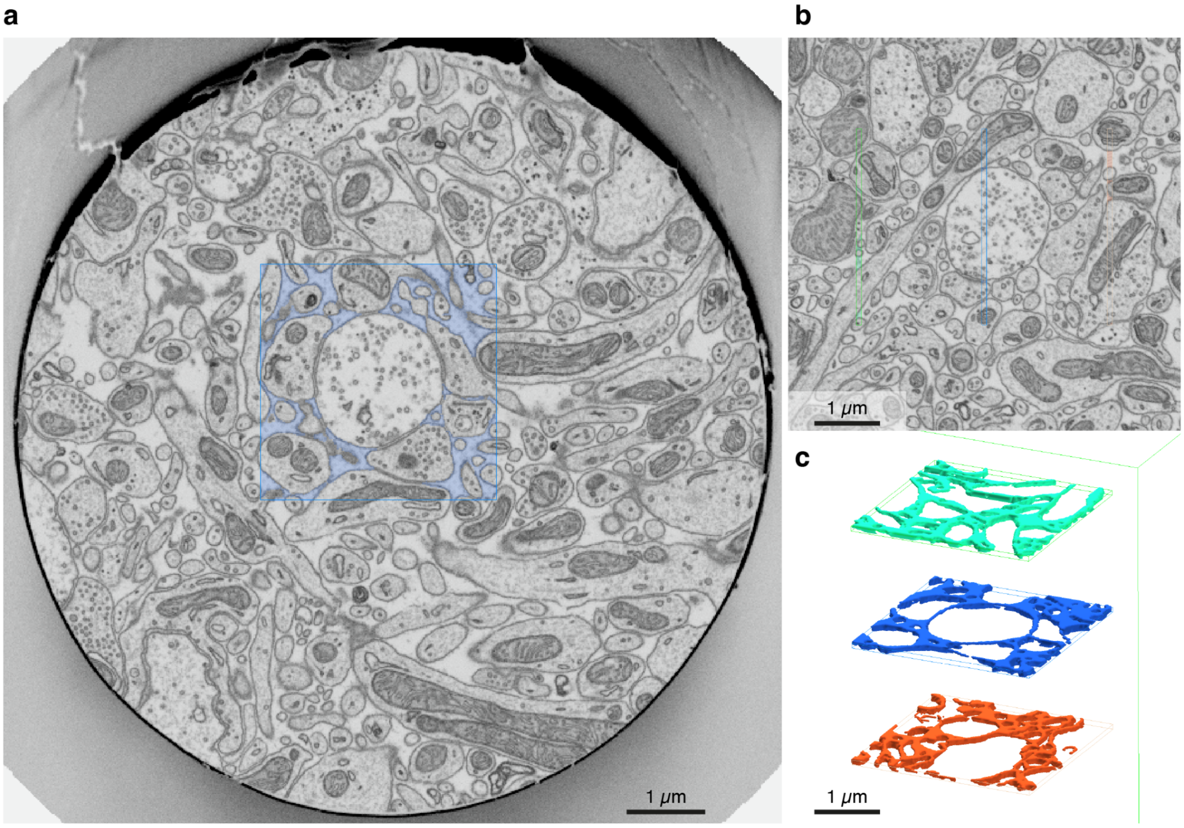

Supplementary Fig. 1: Extracellular space in samples containing olfactory bulb external plexiform layer

(a-b) Orthogonal cross-sections of the FIB-SEM dataset ((8 nm)^3 voxels) obtained after the PXCT experiment from the sample imaged with the best efforts PXCT tomogram. Extracellular space was manually segmented in n=3 regions of interest, each spanning 3x3x0.08 μm^3, located 1.92 μm apart in z.

(c) 3D renders of the extracellular space segmented in the three regions of interest.
