# Supplementary material for: Nondestructive X-ray tomography of brain tissue ultrastructure
Source: Nat Methods. 2025 Nov 27;22(12):2631–8. doi: 10.1038/s41592-025-02891-0 (PMC12695642; doi:10.1038/s41592-025-02891-0)
Supplement: Supplementary file 2 — Reporting Summary [file 41592_2025_2891_MOESM2_ESM.pdf]

nature portfolio

Corresponding author(s): Ana Diaz
Adrian A. Wanner
Andreas T. Schaefer

Last updated by author(s): Aug 28, 2025

Reporting Summary

Nature Portfolio wishes to improve the reproducibility of the work that we publish. This form provides structure for consistency and transparency in reporting. For further information on Nature Portfolio policies, see our Editorial Policies and the Editorial Policy Checklist.

Statistics

For all statistical analyses, confirm that the following items are present in the figure legend, table legend, main text, or Methods section.

n/a	Confirmed	
☐	☒	The exact sample size (*n*) for each experimental group/condition, given as a discrete number and unit of measurement
☐	☒	A statement on whether measurements were taken from distinct samples or whether the same sample was measured repeatedly
☒	☐	The statistical test(s) used AND whether they are one- or two-sided<br>*Only common tests should be described solely by name; describe more complex techniques in the Methods section.*
☒	☐	A description of all covariates tested
☒	☐	A description of any assumptions or corrections, such as tests of normality and adjustment for multiple comparisons
☐	☒	A full description of the statistical parameters including central tendency (e.g. means) or other basic estimates (e.g. regression coefficient) AND variation (e.g. standard deviation) or associated estimates of uncertainty (e.g. confidence intervals)
☒	☐	For null hypothesis testing, the test statistic (e.g. *F*, *t*, *r*) with confidence intervals, effect sizes, degrees of freedom and *P* value noted<br>*Give P values as exact values whenever suitable.*
☒	☐	For Bayesian analysis, information on the choice of priors and Markov chain Monte Carlo settings
☒	☐	For hierarchical and complex designs, identification of the appropriate level for tests and full reporting of outcomes
☒	☐	Estimates of effect sizes (e.g. Cohen's *d*, Pearson's *r*), indicating how they were calculated

Our web collection on statistics for biologists contains articles on many of the points above.

Software and code

Policy information about availability of computer code

Data collection	Most datasets reported in this manuscript were acquired at the cSAXS beamline of the Paul Scherrer Institut (Swiss Light Source). Scattered X-rays were measured by a 2D detector (Eiger 1.5M). For the datasets obtained at BM05 (European Synchrotron), The detector used for imaging was composed of a 23 µm thick LSO scintillator coupled to an infinity-corrected long-working-714 distance Mitutoyo objective (10x, NA 0.28) and a PCO Edge sCMOS camera.
Data analysis	Data processing to reconstruct the X-ray ptychographic reconstructions was performed using custom beamline code in MATLAB at the cSAXS beamline. Datasets acquired at BM05 in ESRF were reconstructed using Nabu (https://tomotools.gitlab-pages.esrf.fr/nabu/about.html). Data analysis was performed using MATLAB (R2022a-R2024b, 9.12.0.1884302). Beamline diagrams were designed with Catia V5 and CorelDRAW (version 25.1.0.269, June 2024 Release). 3D renders were generated with Blender (www.Blender.org; Blender Online Community. (2021). Blender - A 3D modelling and rendering package. Blender Foundation, Stichting Blender Foundation, Amsterdam.)<br><br>Supporting code:<br>https://github.com/cboschp/ptychoStainedTissue<br>Non-destructive X-ray tomography of brain tissue ultrastructure. Supporting code ptychoStainedTissue. (v1.1.0). Zenodo. https://doi.org/10.5281/zenodo.16364654

For manuscripts utilizing custom algorithms or software that are central to the research but not yet described in published literature, software must be made available to editors and reviewers. We strongly encourage code deposition in a community repository (e.g. GitHub). See the Nature Portfolio guidelines for submitting code & software for further information.

Data

Policy information about availability of data

All manuscripts must include a data availability statement. This statement should provide the following information, where applicable:

- Accession codes, unique identifiers, or web links for publicly available datasets
- A description of any restrictions on data availability
- For clinical datasets or third party data, please ensure that the statement adheres to our policy

Source data of the graphs presented in the main figures are provided as a Source Data file. The datasets and major annotations reported in this study are accessible through the associated code repository (see Code Availability).

Supporting dataset:
Non-destructive X-ray tomography of brain tissue ultrastructure. Supporting metadata. [Data set]. Zenodo. https://doi.org/10.5281/zenodo.16362800

Human research participants

Policy information about studies involving human research participants and Sex and Gender in Research.

Reporting on sex and gender	This study did not involve human participants.
Population characteristics	See above.
Recruitment	n/a
Ethics oversight	n/a

Note that full information on the approval of the study protocol must also be provided in the manuscript.

Field-specific reporting

Please select the one below that is the best fit for your research. If you are not sure, read the appropriate sections before making your selection.

☒ Life sciences ☐ Behavioural & social sciences ☐ Ecological, evolutionary & environmental sciences

For a reference copy of the document with all sections, see nature.com/documents/nr-reporting-summary-flat.pdf

Life sciences study design

All studies must disclose on these points even when the disclosure is negative.

Sample size	Tomogram series were defined as a series of consecutive tomograms obtained from the same specimen imaging the same field of view (ie without >2µm variations in sample height) and without any unaccounted exposures that would irradiate >1e6 Gy. This allowed to monitor the accumulated X-ray dose absorbed by the sample across the series. Cylindrical samples of 5-30 µm in width were prepared from mouse brain tissue, targeting whenever possible tissue regions devoid of uninformative 10µm+ large features such as blood vessels or cell nuclei. The external plexiform layer of the olfactory bulb provided a reproducible background within and across individuals, containing a broad range of biological feature sizes (10-1000nm). Sample size was ultimately limited by acquisition (available beamtime) and preparation (FIB availability).
Data exclusions	No datasets were excluded from the analysis.<br>In the synapse detection task, only data series arising from annotators that completed the whole task were kept (n=3 annotators). Within those, cube locations that were missed by any of the three annotators were discarded, leading to a complete response set for 240 locations by 3 annotators that was further analysed.
Replication	We report results arising from 63 tomograms organised in 19 tomogram series acquired from 17 separate samples during 5 beamtimes. Special care was addressed to extend the amount of technical and biological replicates whenever possible.
Randomization	All tomograms were analysed in bulk.
Blinding	Data analysis of pooled tomogram series can be traced to each individual tomogram whenever possible, and individual data points are always shown.

Reporting for specific materials, systems and methods

We require information from authors about some types of materials, experimental systems and methods used in many studies. Here, indicate whether each material, system or method listed is relevant to your study. If you are not sure if a list item applies to your research, read the appropriate section before selecting a response.

Materials & experimental systems

n/a	Involved in the study
☒	☐ Antibodies
☒	☐ Eukaryotic cell lines
☒	☐ Palaeontology and archaeology
☐	☒ Animals and other organisms
☒	☐ Clinical data
☒	☐ Dual use research of concern

Methods

n/a	Involved in the study
☒	☐ ChIP-seq
☒	☐ Flow cytometry
☒	☐ MRI-based neuroimaging

Animals and other research organisms

Policy information about studies involving animals; ARRIVE guidelines recommended for reporting animal research, and Sex and Gender in Research

Laboratory animals	Animals used in this study were 6-16 week old wildtype mice of C57Bl/6 and CD-1 background of either sex. Mice were housed up to 5 per cage with an ad libitum supply of food and water under a 12–12 h light–dark cycle at an ambient temperature of 22±2°C and a relative humidity of 55±10%. All animal IDs are listed in Supp. Table 5.
Wild animals	The study did not involve wild animals.
Reporting on sex	Samples were obtained from mice from either sex. Sex does not suppose any confounding factor in this study. We provide information on the sex and age of all animals related to all samples in the updated Supplementary Table 5.
Field-collected samples	This study did not involve samples collected from the field.
Ethics oversight	All animal protocols applying to all mice except M1 were approved by the Ethics Committee of the board of the Francis Crick Institute and the United Kingdom Home Office under the Animals (Scientific Procedures) Act 1986. For M1, animal experiments were approved by the cantonal veterinary office of the canton Aargau (Switzerland) and were carried out in accordance with the Swiss law on animal protection. All animal IDs are listed in Supp. Table 5.

Note that full information on the approval of the study protocol must also be provided in the manuscript.
